# Cost-Effectiveness of isoniazid preventive therapy among HIV-infected patients clinicaly screened for latent tuberculosis infection in Dar es Salaam, Tanzania: A prospective Cohort study

**DOI:** 10.1186/s12889-017-4597-9

**Published:** 2017-07-19

**Authors:** Grace A. Shayo, Dereck Chitama, Candida Moshiro, Said Aboud, Muhammad Bakari, Ferdinand Mugusi

**Affiliations:** 1 Department of Internal Medicine, Muhimbili University of Health and Allied Sciences, P.O. Box 65001, Dar es Salaam, Tanzania; 2 Department of Development Studies, Muhimbili University of Health and Allied Sciences, P.O. Box 65001, Dar es Salaam, Tanzania; 3 Department of Epidemiology and Biostatistics, Muhimbili University of Health and Allied Sciences, P.O. Box 65001, United Nations road, Dar es Salaam, Tanzania; 4 Department of Microbiology and Immunology, Muhimbili University of Health and Allied Sciences, P.O. Box 65001, United Nations road, Dar es Salaam, Tanzania; 5 Ministry of Health, Community Development, Gender, Elderly and Children, P.O.Box 9083, Mirambo street, Dar es Salaam, Tanzania

**Keywords:** Isoniazid preventive therapy, Cost effectiveness, HIV infection, Clinical algorithm

## Abstract

**Background:**

One of the reasons why Isoniazid preventive therapy (IPT) for Tuberculosis (TB) is not widely used in low income countries is concerns on cost of excluding active TB. We analyzed the cost-effectiveness of IPT provision in Tanzania having ruled out active TB by a symptom-based screening tool.

**Methods:**

Data on IPT cost-effectiveness was prospectively collected from an observational cohort study of 1283 HIV-infected patients on IPT and 1281 controls; followed up for 24 months. The time horizon for the analysis was 2 years. Number of TB cases prevented and deaths averted were used for effectiveness. A micro costing approach was used from a provider perspective. Cost was estimated on the basis of clinical records, market price or interviews with medical staff. We annualized the cost at a discount of 3%. A univariate sensitivity analysis was done. Results are presented in US$ at an average annual exchange rate for the year 2012 which was Tanzania shillings 1562.4 for 1 US $.

**Results:**

The number of TB cases prevented was 420/100,000 persons receiving IPT. The number of deaths averted was 979/100,000 persons receiving IPT. Incremental cost due to IPT provision was US$ 170,490. The incremental cost effective ratio was US $ 405.93 per TB case prevented and US $ 174.15 per death averted. These costs were less than 3 times the 768 US $ Gross Domestic Product (GDP) per capita for Tanzania in the year 2014, making IPT provision after ruling out active TB by the symptom-based screening tool cost-effective. The results were robust to changes in laboratory and radiological tests but not to changes in recurrent, personnel, medication and utility costs.

**Conclusion:**

IPT should be given to HIV-infected patients who screen negative to symptom-based TB screening questionnaire. Its cost-effectiveness supports government policy to integrate IPT to HIV/AIDS care and treatment in the country, given the availability of budget and the capacity of health facilities.

## Background

Isoniazid preventive therapy (IPT) has been found to effectively prevent development of active tuberculosis (TB) in both Human immunodeficiency virus (HIV) infected and uninfected at risk populations [1–4]. Studies have shown that the effectiveness of TB preventive therapies including Isoniazid is more marked among patients with positive tuberculin skin test (TST) than among those with negative TST results [2, 4, 5]. Longer duration of the preventive therapy was found to be more effective than shorter durations. Gupta et al. (2014) found that expanded antiretroviral therapy (ART) at 90% coverage with infectious control (IC) and 36-month IPT averted more TB cases than with increased ART coverage, IC and 6-month IPT among tuberculin skin test positive individuals [6]. Recent studies have specifically showed that IPT reduces all-cause mortality [7, 8], something that was not seen in the pre-ART era [3]. Despite evidence of effectiveness, IPT is not widely provided to HIV infected patients, especially in developing countries. For instance in the year 2013 only 14 of the 41 high HIV/TB burdened countries reported provision of IPT to people living with HIV. Most countries report inability to exclude active TB, an important prerequisite before IPT initiation owing to high costs. Other reasons are unavailability of diagnostics to exclude active TB in HIV-infected patients, fear of INH toxicity and resistance development. To reduce the burden of cost of excluding active TB, WHO recommends the use of a simplified screening algorithm that relies on the absence of all four possible TB clinical symptoms (current cough, night sweats, fever, and weight loss) to identify HIV-infected patients who are less likely to present with active TB disease and thus are eligible for IPT. The clinical screening algorithm is reported to be effective and less costly [9]. This recommendation is mainly for resource limited countries; however it is recommended that TST should be used whenever possible [9]. Some countries have slightly modified the WHO screening tool aiming at making it more sensitive. For instance, Tanzania has a 5-symptom screening tool after adding hemoptysis [10] while Namibia has a 5-symptom screen after adding swollen lymph nodes [11].

Little is known on the cost effectiveness of IPT having screened patients’ eligibility for IPT using symptom-based screening tools. Most studies which have demonstrated cost-effectiveness of IPT in TB prevention had used TST for latent TB screening. For instance, Menzies et al., 2004 demonstrated higher total costs for Isoniazid than for rifampicin preventive therapy [12]. Likewise, a study in India by Pho et al., 2012 found that INH given for 6 months was cost-effective but not ethambutol/isoniazid combination due to high cost of ethambutol [13]. Compared to counterfactual, Bell et al.*,* 1999 demonstrated that when only medical care costs are considered, isoniazid alone or isoniazid in combination with rifampicin or rifampicin in combination with pyrazinamide given for 6 months cost more to extend life and prevent tuberculosis. However, when medical care and social costs were considered together, 6-months of daily INH treatment was found to save money relative to no preventive therapy [14].

This study aimed to evaluate the cost-effectiveness of IPT in patients who initiated IPT having been screened by a clinical algorithm to exclude active TB [15]. We hypothesized that provision of IPT for 6 months to HIV-infected patients having ruled out active TB by a symptom-based clinical algorithm was cost effective compared to not providing IPT. The analysis was performed from the provider perspective as the provision of IPT is expected to be done in both the government and private owned hospitals. The results of this analysis are largely expected to emphasize the need for consideration of providing IPT to HIV-infected patients in Tanzania.

## Methods

### Study sites

This study was a prospective cohort study conducted in six urban-based clinics providing HIV/AIDS treatment services in Dar es Salaam between February 2012 and March 2014. The six HIV care and treatment clinics were Muhimbili National Hospital (MNH), Amana, Temeke, Pastoral Activities and Services for people with AIDS in Dar es Salaam Archdiocese (PASADA), Mwananyamala and Buguruni. All the clinics provided primary HIV care. The clinics at MNH, Amana, Temeke and PASADA were among the initial 14 centers participating in a separate pilot program to evaluate the feasibility of countrywide provision of IPT to HIV-infected patients in Tanzania. We used this opportunity to conduct the present study whereas the four clinics constituted an IPT group. Patients from Mwananyamala and Buguruni facilities were not part of the National IPT pilot program and were thus enrolled to form a control group. All the clinics run for five days per week and each attended about 70-100 patients daily, making a total of 8400-12,000 patients monthly. Patients’ visits in these clinics were scheduled once monthly, bimonthly or trimonthly but participants of the present study were requested to attend monthly.

### Data collection procedures

Detailed methods on data collection procedures are described elsewhere [15, 16]. In brief, this was an observational study nested in the then ongoing pilot study on IPT provision in the country. Patients were consecutively enrolled to receive IPT (IPT group) or did not receive IPT (control group, whose patients came from sites which were not under the pilot study). Inclusion criteria were age ≥ 10 years, HIV-infected, both ARV naïve or experienced and who were willing to stay in Dar es Salaam for at least 2 years. Patients were excluded if they i) were known alcohol abusers (ii) had a history of current or past hepatitis (iii) medical contraindications to IPT, (iv) current or recent (within the past two years) receipt of TB treatment, (v) pregnant (vi) had WHO HIV clinical stage 4 AIDS (vii) had a history of treatment non-compliance [17]. The National Tuberculosis and Leprosy Program (NTLP) symptom-based screening tool was used for screening of active TB. This tool is comprised of 5 questions; presence of cough for ≥2 weeks, fever for ≥2 weeks, hemoptysis of any duration, excessive night sweats for ≥2 weeks and noticeable weight loss, or weight loss of ≥3 kg within 4 weeks [10]. Our previous research among HIV-infected population found that this tool had a sensitivity of 71.4% and a specificity of 75.9% for identifying active TB. Although the false negative rate was as high as 28.6%, the negative predictive value was 98.4% ensuring that about 98% of patients who screen negative to the tool are truly free of active TB. The positive predictive value was 11.4% [18].

Patients who presented with none of the symptoms in the screening tool were declared as not having active TB and were evaluated for IPT eligibility. Presentation of any of the symptoms in the screening tool raised suspicion for active TB and thus patients with symptoms underwent standard TB screening. These were eligible for enrollment upon exclusion of active TB. We defined TB as per the NTLP definition which requires the presence of two of the following: (i) Symptoms of TB (cough, fever, night sweats, loss of weight for more than 2 weeks), (ii) AFB visible by direct Ziehl-Nielsen staining of sputum specimen or *M. tuberculosis* cultured from sputum in Lowenstein–Jensen media, (iii) Chest radiograph independently interpreted as highly suggestive of TB (iv) TB in organs other than the lungs proven by one culture positive specimen from an extra-pulmonary site or histopathological evidence from a biopsy or a strong clinical evidence, including macroscopic evidence of specimen inspection, consistent with active extra-pulmonary TB and the decision by a medical doctor to treat with a full course of anti-tuberculous therapy (v) A clinical response to anti-TB medication in patients with culture- negative TB [17]. For both treatment groups, we collected baseline socio-demographic and clinical data.

### The intervention

Patients in the two groups were under standard care for HIV as per National guidelines [10]. Additionally, patients in the IPT group were provided with a monthly supply of INH 300 mg to be taken orally daily for a total of 6 months. Both groups were followed up monthly for a total of 24 months. Isoniazid (INH) side effects were monitored during treatment.

### Study end points

Primary end points were incident active TB, all-cause mortality, lost to follow-up and transfer out. The secondary end point was death from TB defined as death from any cause except accident related deaths occurring in a patient diagnosed with TB. All-cause mortality was defined as death resulting from any cause after recruitment into the study. Lost to follow-up was defined as absence from the clinic for two consecutive scheduled visits with failure to track the patient. Patients who transferred out were patients who shifted care from an HIV clinic of enrollment to the study to another clinic which was not a study site.

### Effectiveness measurement

We used the number of TB cases prevented and number of deaths averted for the assessment of effectiveness. To diagnose TB, the TB symptom-based screening tool was repeated monthly for the two groups. TB suspects were provided with two 50 mL falcon tubes for sputum collection. Sputum was collected in an open air, on the spot for the 1st specimen and at home on the following morning for the 2nd specimen. Sputum samples were transported immediately to a TB laboratory in the Infectious Diseases Control (IDC) center in the city for *Mycobacterium tuberculosis microscopy* after Ziehl-Nielsen (ZN) staining and for culture in Lowenstein–Jensen (LJ) media. Patients with smear negative results underwent a chest X-ray examination as per NTLP guidelines. All deaths that occurred during follow up were recorded. Assumptions were made that the treatment effect of the IPT was additive to the treatment effect of the ART. Confounding variables to the effects were age, levels of immunosuppression, HIV disease stage, and socioeconomic status of the patients. The treatment effects were presumed to be variable over time as the benefit of IPT wanes off within 2 years of treatment. TB incidence and death rates among patients in the two groups were calculated as the number of TB cases or deaths divided by the total person years of follow-up. Averted TB cases and averted deaths attributable to IPT provision were calculated as the difference between these rates in the control group and the IPT group [19], presented as averted cases or deaths per 100,000 population.

### Cost description and estimation

We chose the provider perspective for the analysis because the provision of IPT would ultimately be done in both the government and private owned hospitals which provides HIV care and treatment. Healthcare costs (cost related to the consumption of resources in the health care system) were obtained from records, government standards and market price and through interviewing respective healthcare workers. We adopted a micro-costing approach which is a cost estimation method that involves a direct enumeration and costing out of every input consumed in the treatment of a particular patient [20, 21]. For the analysis of cost effectiveness we used financial costs (actual monetary expenditures) [21]. Personnel cost was derived from the government salary scales obtained from the office of the Human resources management in the health facilities. Patients in the IPT group could be obtained from 2 clinics only but to hasten recruitment of patients in this arm, 4 clinics were involved. Therefore, in order to avoid spurious inflation of the cost of clinic space and personnel costs in the IPT group, we made an assumption that all the 1283 patients who received IPT were seen in two clinics instead of four to match the two clinics in the control group. For this case, the total number of staff used in cost estimation is that of two clinics in IPT group and two clinics in the control group. Recurrent cost was derived from the sum of the cost for training and that for the clinic space which was estimated as a rented space using the market price for renting 1 square meter per month. All the nurses and doctors in the HIV clinics underwent training on intensified case finding, infection control and Isoniazid Preventive therapy (3Is) organized by the National AIDS Control Program (NACP). The cost data for the training was thus obtained from the NACP. As the training was for 3 days, we assumed each topic used one third of the training days. From this assumption we therefore apportioned 1/3 of the cost of training to IPT training. Prices for INH and other anti-tuberculosis drugs were obtained from the Stop TB Partnership Global Drug Facility (GDF) prices of 2013 [22]. Costs for laboratory tests were taken as an average of the market prices in the year 2012-2013. Other costs considered were salaries for the staff in the HIV clinics (we apportioned the cost to be 10% for IPT services, guided by the average time the staff used to offer IPT-related services to a patient). The costs of investigations to undergo standard screening for tuberculosis were also included (sputum for acid fast bacilli microscopy, full blood count, erythrocyte sedimentation rate and culture in Lowenstein- Jensen media). In this case, number of active TB suspects during follow-up was determined for each group and the total costs of investigations to rule out or diagnose TB was estimated by multiplying the cost of each investigation by the number of suspects. Although for the purpose of this study we had transported sputum for analysis in the Infectious Disease Control (IDC) laboratory, we omitted transportation cost because the facilities had the capacity to process the sputum within their facilities thus the transportation cost was unrealistic to the facilities. For cost of utilities (water and electricity), we apportioned 10% of the expenses in the clinics to IPT services. Except for drug costs which were already in US$, all other costs were estimated in Tanzanian shillings, and converted to US$ using the average annual exchange rate for the year 2012 obtained from the Bank of Tanzania which was Tsh. 1562.4 for 1 US $ [23].

### Data analysis

The cost data was entered into and analyzed with Microsoft excel 2010. The analysis was annualized in the 1st and 2nd year and a discount of 3% was applied to the cost of the 2nd year. The time horizon for the analysis was 2 years. ICER was calculated as the difference of the total cost for each group divided by the difference in the effectiveness (TB cases prevented and deaths averted). A univariate sensitivity analysis of cost was performed and it involved analysis of one cost parameter after another when others were kept constant. A sensitivity range of +/−10% was used to vary the base case values of the incremental cost parameters for the entire cohort while observing the change in the ICER per TB case prevented and per deaths averted. The choice of the sensitivity range was guided by an average annual inflation rate which is around 7.31% from 1999 until 2017 [24]. There were five cost categories analyzed; personnel, laboratory, medication, utilities and recurrent costs. We also checked the robustness of the effects, number of TB cases prevented by IPT and that of deaths averted by IPT on the ICER. Patients’ characteristics and outcomes were analyzed using SPSS statistical package version 20. Baseline characteristics were analyzed using proportions, medians ± interquartile range (IQR) and means ± standard deviation (± SD). Chi square was used to compare proportions, student t-test to compare means while medians were compared using K independent samples.

## Results

A total of 2564 patients were followed up for 24 months. One thousand two hundred eighty-three patients received IPT for the first six months (IPT group) and 1281 controls did not receive IPT. Table 1 shows patients characteristics in the two groups. The majority of patients were female (76%). Patients in the IPT group were significantly older than those in the control group (*p* < 0.001), had higher median BMI (*p* < 0.001), had significantly higher baseline CD4 counts (*p* < 0.001), had significant more patients on ART (*p* = 0.025), and had been on ART for a longer period than those in the control group (*p* < 0.001), (Table 1).

**Table 1:** Patients’ characteristics and outcomes

Characteristic	All patients *N* = 2564	IPT group *N* = 1283	Control group *N* = 1281	*P* value
Sex: Females [Number (%)]	1949 (76.0)	994(77.4)	955(74.6)	0.083
Mean age in years (±SD)	38.6(10.3)	39.4(±10.5)	37.8(±10.1)	<0.001
Median BMI (IQR)	22.8(7)	23.1(6)	22.7(6)	<0.001
Patients on ARV drugs [Number (%)]	1913(75)	982(76.5)	931(72.7)	0.025
Median ARV duration in months(IQR) ^a^N=1893	28.0(37)	31.0(38)	23(36)	<0.001
Mean baseline CD4+ cells/μl (± SD), ^b^N=2357	375 (±247)	415(±261)	330(±223)	<0.001
Patients completing 6 months of IPT [N (%)]	1263(98)	1263(98)	-	NA
Patients developed TB during follow up [N (%)]	13 (1.1)	2 (0.2)	11 (0.9)	0.012
Number of deaths during follow up [N (%)]	27 (0.5)	3(0.2)	24(1.9)	<0.001
Patients lost to follow up [N (%)]	146(5.7)	73(5.7)	73(5.7)	0.992
Patients transferred out [N (%)]	110(4.3)	31(2.4)	79(6.2)	<0.001
Patients who withdrew consent [N (%)]	13(0.5)	10(0.8)	3(0.2)	0.052
Patients who completed study	2268(88.5)	1166 (90.9)	1102 (86.0)	<0.001

*NA* Not applicable

^a^Only 1893 were on ARV treatment

^b^Only 2357 patients had known baseline CD4 counts. Baseline here refers to the CD4 value at the time of study recruitment

Significantly more patients from the control group developed active TB (11/1281), died of any cause (24/1281), and were transferred out than were patients from the IPT, (*p* values 0.012, <0.001 and <0.001 respectively). Significantly more patients (10/1283) in the IPT group withdrew consent to continue with the study as compared to patients in the Control group (3/2081). There was equal number of loss to follow up in the two groups (Table 1).

Table 2 shows effectiveness data. A total of 420 TB cases per 100,000 population were prevented and a total of 979 deaths per 100,000 population were averted through IPT use.

**Table 2:** Effectiveness data for the study

	IPT group	Control group	Number of cases averted per 100,000 persons
Person years of follow up (PY)	2205	2152	
TB incidence density/100,000 PY	91	511	420
Mortality incidence density/100,000 PY	136	1115	979

Table 3 shows unit costs in US $ for the cost parameters used as inputs in the cost effectiveness analysis.

**Table 3:** Unit costs in US $ for the cost parameters used in the cost-effectiveness analysis

Cost parameter	Salary scale	Number in place		Unit cost (US $)
		IPT	Controls	
Personnel costs				
Assistant medical officer	TGHS C	6	6	567.64
Nurse	TGHS B	1	0	278.37
Health attendant	PMOSS 1/1	2	2	391.25
Assistant nurse	TGHS A	16	16	300.81
Nurse counsellors	PMGSS 4/1	8	8	727.98
Medical doctors	PMGSS 8/1	4	4	1309.09
Medical specialists	PMGSS 11/1	4	4	2418.00
Laboratory scientific officer	PMGSS 6/1	4	4	982.62
Laboratory technician	PMGSS 4/1	4	4	727.98
Pharmacist	PMGSS 6/1	4	4	982.62
Pharmacy technician	PMGSS 4/3	4	4	727.98
Laboratory costs				
Sputum microscopy		23	21	1.92
Sputum culture		23	21	9.62
FBP		23	21	3.84
ESR		23	21	1.28
CXR		23	21	12.83
Average water bill per month		N/A	N/A	295.04
Average electricity bill per month		N/A	N/A	577.26
Estimated average clinic area (square metres)		256	256	18^a^
Number Trained on 3Is for 3 days		33	32	358.80

*N/A* not applicable

^a^Renting price per square meter per month

Table 4 shows annualized estimated cost figures used in each ingredient of the cost effectiveness analysis for both groups. Majority of the cost came from personnel costs. The annual cost of INH was US **$** 94,807. Total cost of providing IPT was US **$** 1,417,273.29 while total cost in the controls was US $1,246,783.28. The incremental cost due to IPT provision for the entire cohort was US$ 170,490.00. The Incremental cost effectiveness ratio (ICER) was US $ 405.93 per TB case prevented and US $174.15 per death averted (Table 4).

**Table 4:** Cost figures used in the cost analysis of Isoniazid Preventive Therapy for HIV-infected patients in Dar es Salaam for 24 months of follow up^a^

Description Cost parameter	Cost in IPT group (US $)			Cost in the Control group (US $)		
	1st year	2nd year (3% discount)	Total cost for 2 years	1st year	2nd year (3% discount)	Total cost for 2 years
Clinic personnel						
Doctors	225,317	218,754	444,071	216,074	213,057	429,131
Nurses	197,738	191,978	389,716	175,074	169,974	345,048
Medical assistants	44,890	43,583	88,473	40,810	39,621	80,431
Laboratory staff	82,808	80,396	163,204	81,988	79,600	161,588
Pharmacy staff	82,808	80,396	163,204	81,988	79,600	161,588
Laboratory tests						
Sputum smear	27	17	44	21	19	40
Sputum culture	134	86	220	106	96	202
Chest X-ray	179	115	294	141	128	269
Full blood count	54	35	89	42	38	81
ESR	18	12	30	14	13	27
Medication costs						
INH (300 mg/day)^b^	94,807	-	94, 807	-	-	-
1st line anti-TB drugs^c^	265	-	265	927	514	1441
Utilities						
Water	3885	3773	7658	3533	3430	6963
Electricity	15,207	14,764	29,971	13,825	13,422	27,247
Recurrent costs						0
Clinic space	60,826	59,054	119,880	55,296	53,685	108,981
Personnel training	3819	-	3819	4177	-	4177
Total cost	767,892	649,382	1,417,273	633,205	613,578	1,246,783
Incremental cost^d^			US$ 170,490			
ICER per TB case prevented^e^			US$ 405.93 per TB case prevented			
ICER per death averted^f^			US$ 174.15 per death averted			

*IPT* Isoniazid preventive therapy, HIV-Human immunodeficiency virus, INH- Isoniazid, TB-Tuberculosis

*IT* Information technology, ESR -Erythrocyte sedimentation rate

^a^Annualized total costs for the two groups ^b^INH for IPT group only

^c^1st line anti-TB drugs were given to patients who developed active tuberculosis

^d^Discounted cost for 2 years in the IPT group minus discounted cost for 2 years in the Control group

^e^Incremental cost for the entire cohort divided by number of TB cases prevented

^f^Incremental cost for the entire cohort divided by number of deaths averted

The incremental cost per person to start IPT was US $ 132.88. At an observed mean adherence rate of 98.9% from a previous published paper from the same cohort [16], the incremental cost per person to complete 6 months of IPT was US $ 134.99 (Table 5).

**Table 5:** Outcomes and costs of IPT (in US $) among HIV-infected patients in Dar-es Salaam

	All patients	IPT group	Controls
Number of enrolled patients in the two groups	2564	1283	1281
Number of patients Completing 6 months of IPT	1255	1255	N/A
Number of patients developed active TB during follow up	13	2	11
Number of deaths occurred during follow up	27	3	24
Estimated IPT effectiveness using incident TB^*a*^	420	N/A	N/A
Estimated IPT effectiveness using all-cause mortality^*b*^	979	N/A	N/A
Cost per person starting IPT^*c*^ US $	N/A	132.88	N/A
Cost per person to complete 6 months of IPT^*d*^ US $	N/A	135.85	N/A

*TB* Tuberculosis

*N/A* Not applicable

^a^Number of TB cases prevented per 100,000 population

^b^Number of all-cause mortality averted per 100,000 population

^c^Incremental cost due to IPT divided by the number of patients enrolling in IPT

^d^Incremental cost due to IPT divided by the number of patients completing 6 months of IPT

Sensitivity analysis to examine the impact of changes in cost and program effectiveness revealed that the cost per TB case prevented and the cost per death averted through IPT were robust to changes in laboratory and radiological tests but were sensitive to changes in recurrent, personnel, medication and utility costs. Using a sensitivity range of +/−10%, the ICER ranged from 398.91 to 429.44US $ per TB case prevented and from 167.73 to 180.57 US $ per death averted when the incremental personnel cost was varied from 56,557 to 69,125 US $. The ICER ranged from 383.64 to 428.22 US $ per TB case prevented and from 164.58 to 183.71 US $ per death averted when the incremental medication cost was varied from 84,267 to 102,993 US $. The ICER ranged from 403.42 to 408.44 US $ per TB case prevented and from 173.07 to 175.22 US $ per death averted when the incremental recurrent cost was varied from 9486 to 11,594 US $. When TB cases prevented were varied from 378 to 462 per 100,000 persons the ICER ranged from 451.03 to 369.03 US $ per TB case prevented. When deaths averted were varied from 881 to 1077 deaths per 100,000 persons the ICER ranged from 193.52 to 158.30 US $ per death averted, respectively (Table 6).

**Table 6:** Results of the univariate sensitivity analysis for the cost parameters and effectiveness indicators

Using +/− 10% sensitivity range			ICER per TB case prevented (US $)		ICER per death averted (US $)	
Parameter	Sensitivity range (+/−10%)	Sensitivity value	With Low value	With High value	With Low value	With High value
1. Personnel cost	Low	56,557	398.91		167.73	
	High	69,125		429.44		180.57
2. Laboratory cost	Low	53	405.91		174.14	
	High	65		405.94		174.15
3. Medication cost	Low	84,267	383.64		164.58	
	High	102,993		428.22		183.71
4. Utilities cost	Low	3078	405.11		173.80	
	High	3762		406.74		174.50
5. Recurrent cost	Low	9486	403.42		173.07	
	High	11,594		408.44		175.22
6. TB cases prevented	Low	378	451.03			
	High	462		369.03		
7. Deaths averted	Low	881			193.52	
	High	1077				158.30

## Discussion

In the present study, the provision of IPT to HIV-infected patients having excluded active TB using the symptom-based screening tool was found to be cost-effective. This finding has yet to be confirmed in other studies as to our knowledge this is the first study to assess the cost-effectiveness of IPT having excluded active TB by the symptom-based screening tool only. Prior studies have demonstrated that IPT is cost-effective when TST screening strategy was used [25, 26]. Smith et al. have further demonstrated that provision of IPT to HIV-infected TST positive patients was more effective and costly when IPT was given for 36 months than when given for 6 months (which was also cost-effective) [26]. The cost per person starting IPT was no different from the cost per person completing 6 months of IPT. This finding is due to the fact that a large percentage of the patients (97.8%) completed the 6 months of IPT and medication adherence was high (98.9%) [16] thus there was no big difference in the number of patients starting IPT and the number completing IPT. The cost to start IPT is comparable to the cost of starting IPT in Battambang Cambodia of US$ 105 in the year 2003-2006 [25], taking into consideration the difference in time between the two studies. The Cambodian study however came from a meta-analysis [5] in which tuberculin skin test (TST) was used to identify those with latent TB infection.

More than half (55.6%) of the incremental cost for IPT provision came from the cost of INH tablets alone, meaning that only a small portion of the cost came from personnel, laboratory and recurrent costs. This emphasizes the need for integration of IPT provision to HIV services. A univariate sensitivity analysis showed that variation of incremental personnel, recurrent, medication and utility costs invariably changed the ICER per TB case prevented and per death averted but not variations of incremental cost due to laboratory and radiological tests. This is probably due to the fact that laboratory investigations are done sparingly when a symptom-based screening tool is used. This finding might not be the case when TST is considered for identification of latent TB infection. Sutton et al. [26], whose analysis included the use of TST found that the cost of intensified case finding (ICF) per TB case detected and that of IPT per TB case prevented were sensitive to changes in the cost of diagnostic tests [25].

Likewise, incremental cost effective ratios varied with variations in the effects (number of TB cases prevented per 100,000 persons and number of deaths averted per 100,000 persons completing IPT). The incremental cost effectiveness ratio per TB case prevented through IPT (US 405.93) and the incremental cost per death averted through IPT (US $ 174.15) are less than 3 times the US $768 Gross Domestic Product (GDP) per capita for Tanzania in the year 2014 [24], making IPT cost-effective [27, 28]. Although the intervention is cost-effective, it might not be affordable by the healthcare providers owing to constrained health budgets.

### Study limitations

Effectiveness data used in this analysis came from a cohort study with heterogeneous patients in the two treatment groups, which might have contributed to the difference in effectiveness data observed. However, the sample size was large enough to reach significant levels even in very slight differences in socio-demographic and clinical data. Furthermore, in the same study population Cox proportional hazard analysis found that none of the baseline characteristics could predict an occurrence of tuberculosis while ARV use for 2 or more years and IPT use predicted all-cause mortality [15]. As the screening of patients was through the use of a symptom-based screening tool, care should be taken in comparing the findings of this study to similar studies which used TST for screening.

## Conclusions

IPT provision to HIV-infected patients after ruling out active TB using the symptom-based screening tool is cost-effective. The cost per person to start IPT was similar to the cost per person to complete six months of IPT owing to high adherence and treatment completion rates. The largest portion of the incremental cost due to IPT provision came from INH medication alone. We therefore recommend that whenever affordable, IPT should be given to HIV-infected patients who screen negative to symptom-based TB screening questionnaire. Its cost-effectiveness supports government policy for TB/HIV collaborative services, given the availability of budget and health facilities’ capacity.

## Abbreviations

AIDS: Acquired immunodeficiency syndrome
ART: Antiretroviral therapy
ARV: Antiretroviral drugs
CD4: Cluster of differentiation 4
CTC: Care and treatment center
GDF: Global drug facility
GDP: Gross domestic product
HIV: Human immunodeficiency virus
IDC: Infectious disease control
INH: Isoniazid
IPT: Isoniazid preventive therapy
IQR: Interquartile range
LJ: Lowenstain-Jensen culture media
MNH: Muhimbili National Hospital
NACP: National AIDS control program
NTLP: National tuberculosis and leprosy program
PASADA: Pastoral Activities and Services for people with AIDS in Dar es Salaam Archdiocese
SD: Standard deviation
TB: Tuberculosis
TST: Tuberculin skin test
US: United States of America
WHO: World health organization
ZN: Ziehl-Nielsen stain
